# Glycosylated nanostructures in sublingual immunotherapy induce long-lasting tolerance in LTP allergy mouse model

**DOI:** 10.1038/s41598-019-40114-7

**Published:** 2019-03-11

**Authors:** Maria J. Rodriguez, Javier Ramos-Soriano, James R. Perkins, Ainhoa Mascaraque, Maria J. Torres, Francisca Gomez, Araceli Diaz-Perales, Javier Rojo, Cristobalina Mayorga

**Affiliations:** 1 Allergy Research Group, Instituto de Investigación Biomédica de Málaga-IBIMA, Málaga, Spain; 2 Medicine Department, Universidad de Málaga-UMA, Málaga, Spain; 3 Glycosystems Laboratory, Instituto de Investigaciones Químicas (IIQ), CSIC - Universidad de Sevilla, Sevilla, Spain; 4 Allergy Clinical Unit, Hospital Regional Universitario de Málaga, Málaga, Spain; 5 Nanostructures for Diagnosing and Treatment of Allergic Diseases Laboratory, Centro Andaluz de Nanomedicina y Biotecnología-BIONAND, Málaga, Spain; 6 Center for Plant Biotechnology and Genomic (UPM-INIA), Madrid, Spain

## Abstract

An effective specific immunotherapy should contain elements to generate specific recognition (T-cell peptides) and to modulate the immunological response towards a Th1/Treg pattern by enhancing dendritic cells (DCs). We propose a novel sublingual immunotherapy for peach allergy, using systems, that combine Prup3-T-cell peptides with mannose dendrons (D_1_ManPrup3 and D_4_ManPrup3). Peach anaphylactic mice were treated 1, 2 and 5 nM concentrations. Tolerance was assessed one/five weeks after finishing treatment by determining *in vivo*/*in vitro* parameters after challenge with Prup3. Only mice receiving D_1_ManPrup3 at 2 nM were protected from anaphylaxis (no temperature changes, decrease in Prup3-sIgE and -sIgG1 antibody levels, and secreting cells) compared to PBS-treated mice. Moreover, an increase of Treg-cells and regulatory cytokines (IL-10^+^/IFN-γ^+^) in CD4^+^-T-cells and DCs were found. These changes were maintained at least five weeks after stopping treatment. D_1_ManPrup3 is an effective new approach of immunotherapy inducing protection from anaphylaxis which persists after finishing treatment.

## Introduction

The increasing prevalence of food allergy (FA) in developed countries, represents a heavy burden for health systems, affecting around 8% of children and 5% of adults^1^. FA is often induced by fruits, especially members of the Rosaceae family, such as peach and apple. The major allergens from this family are non-specific lipid transfer proteins (nsLTP), considered panallergens. Reactions present a complex clinical pattern, and a wide range of cross-reactivity to food derived from multiple sources, known as LTP syndrome^2^.

Avoidance of the causal food is the first-line approach for managing FA. However, accidental ingestion is often inevitable due to the ubiquity of panallergens and hidden sources^3^, strongly limiting quality of life, which has been described in one study of children as worse than for chronic diseases like diabetes^4^. Therefore, there is a clear need for safe and effective treatment.

One such approach is allergen specific immunotherapy (AIT). Clinical AIT studies have been performed for peanut, milk, hazelnut and kiwi^5^, however there is a risk of anaphylactic side effects during treatment with natural allergens^6^. For LTP allergy, sublingual immunotherapy (SLIT) with the major peach allergen, Prup3, produces clinically beneficial effects for mild, moderate, and even severe reactions^7–10^. Studies of the immunological events associated with the tolerant response induced by SLIT have found increases of allergen-specific IgG4 (sIgG4) and decreases of allergen-specific IgE (sIgE) and effector T cells (Th2 and Th9). These changes are orchestrated by dendritic cell (DCs) that modulate the response towards a Th1 pattern, and the generation of FoxP3^+^ regulatory T cells (Treg), which develop their suppressive activity mainly by the induction of IL-10^+^ Treg cells^9–11^.

Nevertheless, AIT does not fully reduce the risk of severe reactions^5^. The international consensus consortium on AIT has suggested approaches to improve the efficacy of AIT, such as allergen-derived peptides^12^ that lack the capacity to cross-link IgE or activate effector cells, making them safer, as demonstrated for house dust mites (HDM), grass^13^ and bee venom allergies^14^. However, recent trials, including a phase III trial with cat peptide AIT and a phase IIb trial with HDM peptide AIT, did not achieve their clinical end-points^13^ showing the need for further improvements.

One approach under study is combining the T-cell peptide with a system to induce appropriate antigen presentation by DCs. We have previously demonstrated that SLIT with a system combining Prup3 T-cell peptides and an oligodeoxyribonucleotide sequence with CpG motifs, intended to act as an adjuvant through TLR9 interaction, induces a specific Th1/Treg response producing protection from anaphylaxis^15^. Similar results were obtained by Srivastava *et al*., combining CpG coated nanoparticles with peanut extract^16^.

Targeting DCs can be further fine-tuned by altering the properties of the nanostructure, i.e. size/carbohydrate composition, to facilitate antigen uptake and presentation by DCs^17^. For example, glycosystems attaching of mannose to nanocarriers have been shown to improve uptake efficiency by targeting a DC C-type lectin receptor, affecting immunomodulation^18,19^.

In this study, we propose the use of glycodendropeptides (GDPs) to improve SLIT for treating FA. These GDPs include a Prup3 peptide, either monovalent (D_1_ManPrup3) or tetravalent (D_4_ManPrup3), bound to a mannose dendron structure. Results show that the monovalent molecule, D_1_ManPrup3 leads to protection upon allergen exposure, with no signs of anaphylaxis. Protection was maintained for at least five weeks after treatment and was associated with a reduced Th2 response (IgE and IgG1) and increased Th1/Treg (IFN-γ and IL-10).

## Results

### Synthesis of GDP nanostructures conjugated with Prup3 T-cell epitope

**Figure 1:**
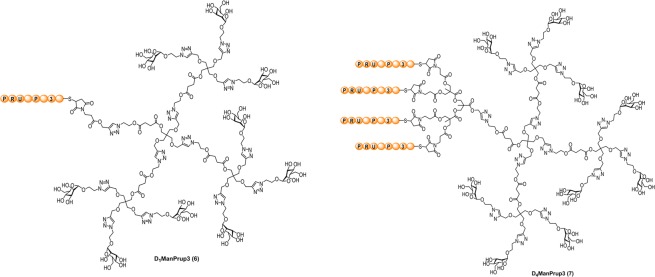
Schematic structures of multivalent glycodendrimers with Prup3 T cell epitopes as monovalent, D_1_ManPrup3 and tetravalent, D_4_ManPrup3.

Synthesis of D_1_ManPrup3 and D_4_ManPrup3 (Fig. 1) was achieved using the strategy described in the Supplementary Material (See Fig. S1). We have previously prepared GDPs with different peptide epitopes^20^ using a similar approach. The glycodendritic platform contains a glycodendron moiety at one site and either 1 or 4 copies of a maleimide group at the other site. The glyco-maleimide dendrons can be used as common intermediates to be conjugated with any type of peptidic epitopes, highlighting the potential versatility of this approach. Here, GDPs D_1_ManPrup3 (**6**) and D_4_ManPrup3 (**7**) were prepared in good yields of high purity as demonstrated by HPLC and mass spectrometry (see Supplementary Material).

### Analysis of the *in vivo* response to Prup3 after receiving SLIT with D_n_ManPrup3

**Figure 2:**
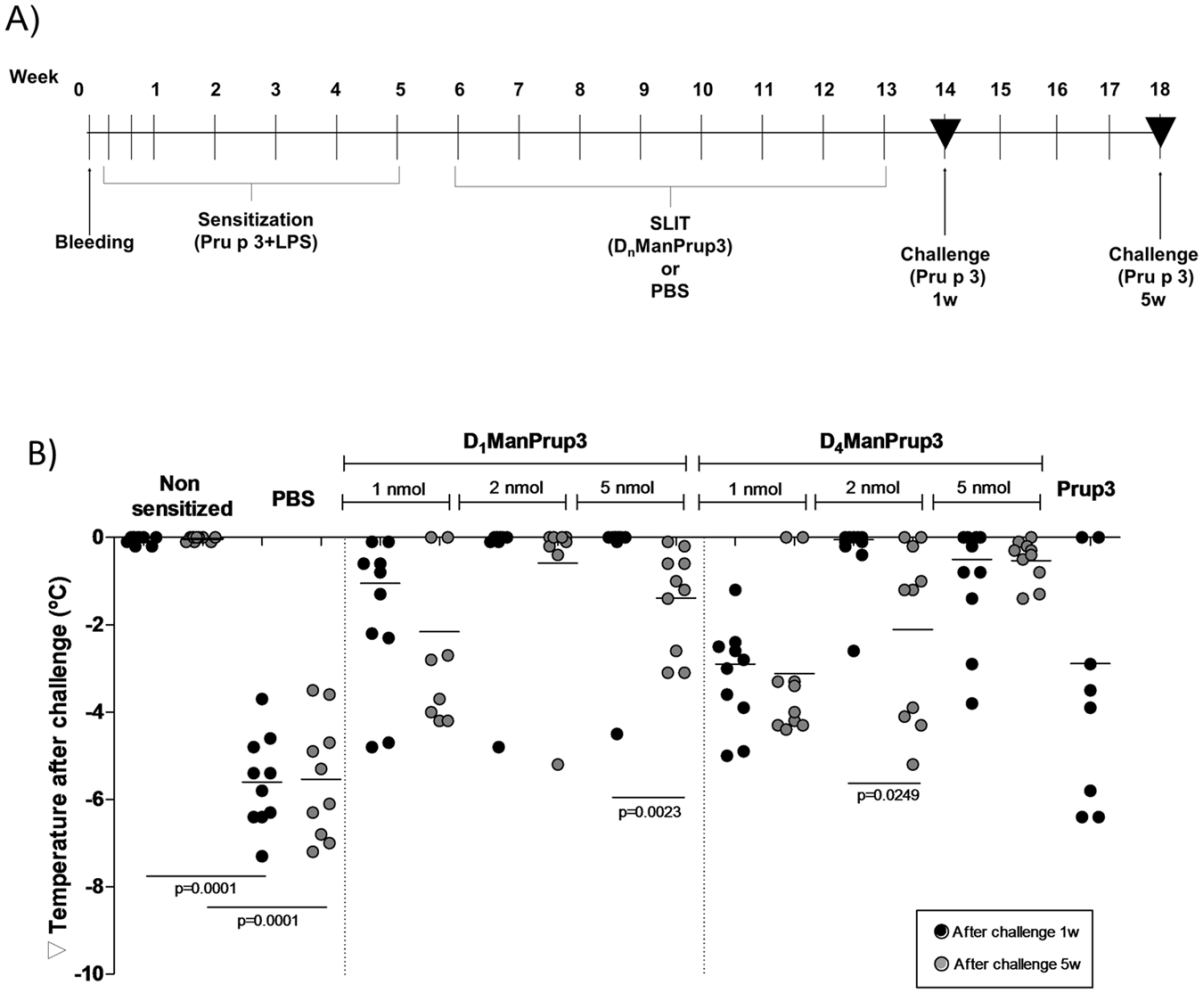
(**A**) Experimental procedures. Seven sensitization procedures were performed with Prup3 + LPS. SLIT started at week 6 for 8 weeks and mice were challenged with Prup3 at week 14 or 18. (**B**) *In vivo* evaluation of anaphylaxis after SLIT with D_1_ManPrup3 or D_4_ManPrup3. Dots show decreases in body temperature 30–40 min after challenge with Prup3, 1 week (black dots) or 5 weeks after ending the SLIT.

The SLIT with D_n_ManPrup3 was safe and no mice showed any adverse effect during the treatment. The *in vivo* response was analyzed by change in body temperature before and after challenge with intraperitoneal Prup3 (Fig. 2B) either one or five weeks after SLIT, with no change indicating tolerance. All GDP treated animals showed a smaller drop in temperature than PBS treated, at both one and five weeks. Mice treated with 2 nM or 5 nM of either D_1_ManPrup3 or D_4_ManPrup3 showed little or no reduction in temperature upon challenge, similar to the non-sensitized mice, at one week after finishing SLIT, although a few of the D_4_ManPrup3 treated mice showed some decrease, as described below. 5 w after finishing, this lack of decrease after challenge was maintained for mice treated with D_1_ManPrup3 at 2 nM and D_4_ManPrup3 at 5 nM. The non-sensitized control group showed no change in temperature after challenge at any time point. The analysis of the response in group 17, treated with Prup3, indicated a similar decrease in the body temperature compared to the PBS treated group and that tolerance was only achieved in two out of ten mice (20%).

Nine mice (90%) treated with SLIT D_1_ManPrup3 at 2 nM and 5 nM and D_4_ManPrup3 at 2 nM maintained their body temperature without systemic symptoms, much higher than for mice treated with SLIT D_1_ManPrup3 at 1 nM (2 mice, 20%) and D_4_ManPrup3 at 1 nM (0 mice) and 5 nM (5 mice, 50%). Moreover, after 5 weeks without treatment, only the group treated with SLIT D_1_ManPrup3 at 2 nM contained a high percentage of mice (90%) showing no changes in body temperature.

### Analysis of the humoral response to Prup3 after receiving SLIT with D_n_ManPrup3

Assessment of serum specific antibody levels (Table 1) showed a decrease in Prup3-sIgE and sIgG1 in GDP treated mice compared to those treated with PBS, with the exception of those treated with D_4_ManPrup3 at 2 nM/5 nM for IgE and D_4_ManPrup3 5 nM for IgG1. Decreases were also observed for the number of Prup3-sIgE and -sIgG1-secreting cells as determined by ELISpot. We also compared antibody levels between those observed 5 w after finishing treatment and 1 w. This revealed a significant increase of Prup3-sIgE in those treated with D_1_ManPrup3 and D_4_ManPrup3 at 5 nM (p = 0.001 for both groups), showing similar levels compared to the PBS-treated group at 5 w. Regarding secreting cell numbers, an increase was observed for mice treated with D_1_ManPrup3 at 1 nM. No changes were observed in Prup3-sIgE and sIgG1 in Prup3 treated mice compared to those treated with PBS although significant decrease of Prup3-sIgE and -sIgG1-secreting cells (p = 0.017 and p = 0.0016, respectively) were found.

**Table 1:** Prup3-specific IgE and IgG1 levels in serum and numbers of secreting cells.

Group		IgE						IgG1					
		ELISA			ELISPOT			ELISA			ELISPOT		
		1 w	5 w	P value	1 w	5 w	P value	1 w	5 w	P value	1 w	5 w	P value
Non-sensitized		0,054 ± 0,003*			27,1 ± 7,4*			0,057 ± 0,01*			16,7 ± 7,1*		
PBS		0,08 ± 0,01	0,08 ± 0,01		120,7 ± 26,3	118,7 ± 30		2,28 ± 0,06	2,31 ± 0,11		108,4 ± 21,3	105,6 ± 26,2	
Prup3		0,08 ± 0,02			66,8 ± 47,3*		0,017	2,10 ± 0,71			51 ± 31,8*		0,0016
D_1_Man Prup3	1nmol	0,06 ± 0,01*	0,06 ± 0,00*	0,10	19,9 ± 17,5*	80,5 ± 27,5*	**0,000**	1,59 ± 0,16*	1,27 ± 0,71*	0,54	53,2 ± 14,7*	80,4 ± 21,8*	**0,01**
	2nmol	0,06 ± 0,00*	0,07 ± 0,02*	0,24	44,5 ± 2,5*	46,5 ± 29,4*	0,62	0,52 ± 0,68*	0,249 ± 0,37*	0,21	43,4 ± 29.1*	38,4 ± 22,2*	0,85
	5nmol	0,05 ± 0,00*	0,07 ± 0,01*	**0,001**	49,9 ± 20,0*	73,3 ± 34,8*	0,10	0,66 ± 0,53*	0,37 ± 0,57*	0,16	34,0 ± 30,9*	55,2 ± 36,1*	0,13
D_4_Man Prup3	1nmol	0,07 ± 0,01*	0,07 ± 0,01*	0,27	81,2 ± 36,8*	113,7 ± 59,6*	0,50	1,54 ± 0,31*	1,74 ± 0,08*	0,28	68,0 ± 26,2*	79,6 ± 43,9*	0,97
	2nmol	0,06 ± 0,03*	0,06 ± 0,04	0,91	57,7 ± 38,8*	92,4 ± 40,5*	0,07	0,61 ± 0,47*	1,12 ± 0,84*	0,32	45,9 ± 30,4*	87,1 ± 55,0*	0,06
	5nmol	0,03 ± 0,02*	0,08 ± 0,02	**0,001**	50,4 ± 26,1*	55,4 ± 18,3	0,27	0,65 ± 0,53*	1,57 ± 0,97	0,07	64,1 ± 43,6*	56,3 ± 27,2	0,62

*Represents significant differences (p < 0.05) when compared with PBS treated mice. P-values represent comparisons between 1 w and 5 w for each treatment.

### Analysis of the cellular response to Prup3 after receiving SLIT

**Figure 3:**
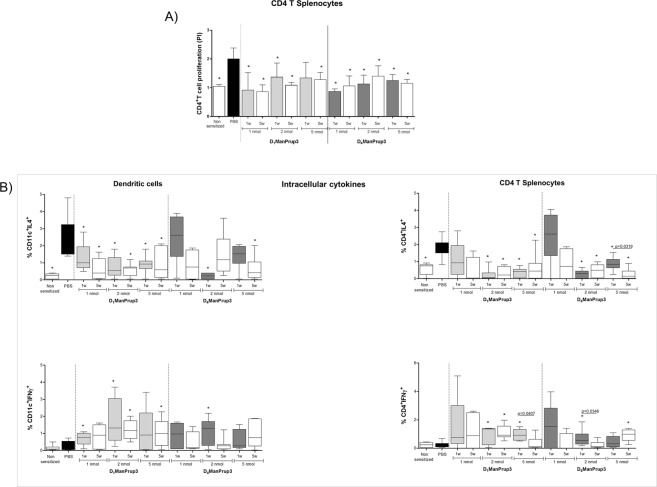
Evaluation of the cellular response to Prup3 after SLIT with D_1_ManPrup3 or D_4_ManPrup3. (**A**) Mean and S.D. Prup3-specific CD4^+^T-splenocytes proliferation index. Statistically significant differences (p < 0.05) compared to PBS-treated represented as *. (**B**) Median and interquartile ranges for CD11c^+^ (Left) and CD4^+^-cell percentages (Right) producing intracellular IL-4/IFN-γ. Significant differences (p < 0.05) compared to PBS treated group represented as *.

There was a significant decrease in CD4^+^ T splenocyte proliferation to Prup3 in GDP-treated mice compared to the PBS-treated group, with the exception of D_1_ManPrup3 5 nM at 1 w (Fig. 3A).

We evaluated the intracellular production of IL-4 and IFN-γ in DCs from lymph nodes and splenocytes after Prup3 stimulation (Fig. 3B). In DCs, a decrease in IL-4-producing CD11c^+^-cells was observed for all mice treated with D_1_ManPrup3 at all concentrations at both 1 w and 5 w post treatment, and with D_4_ManPrup3 at 2 nM, compared to the PBS-treated group. In contrast, an increase of IFN-γ-producing CD11c^+^-cells only occurred in animals treated with at 1 and 2 nM of D_1_ManPrup3 at 1 w, and 2 nM of D_4_ManPrup3 at 1 w. An increase was also observed for 2 and 5 nM of D_1_ManPrup3 at 5 w (Fig. 3B left).

In CD4^+^ cells, a significant decrease in IL-4-producing cells was observed in mice treated with either GDP at 2 and 5 nM compared to PBS-treated group, at both 1 w and 5 w. Moreover, an increase in IFN-γ-producing CD4^+^-cells was observed in mice treated with D_1_ManPrup3 at 2 and 5 nM at 1 w, and for 2 nM at 1 w; and with D_4_ManPrup3 at 2 nM at 1 w, and 5 nM at 5 w (Fig. 3B, right).

### Analysis of the regulatory response to Prup3 after receiving SLIT

**Figure 4:**
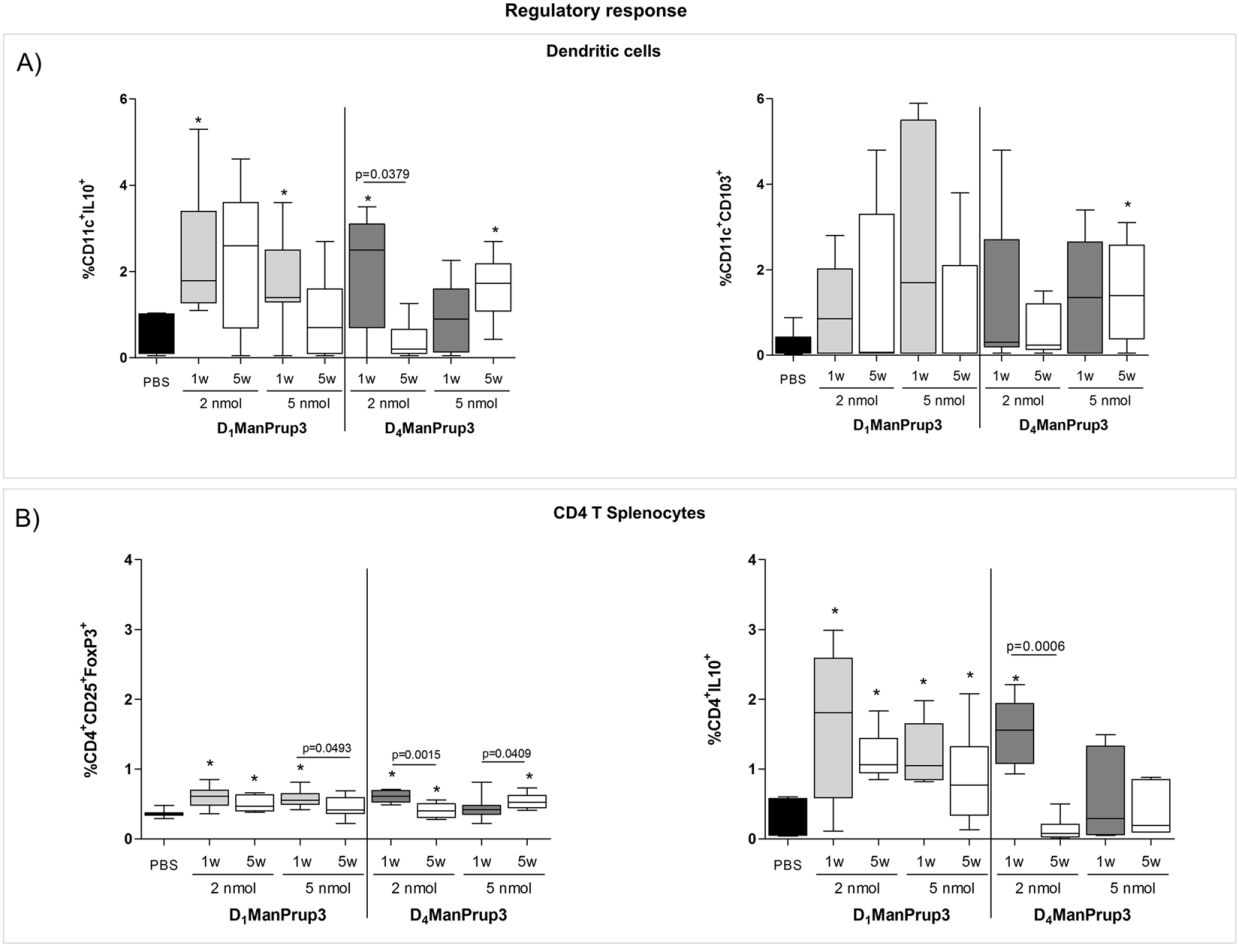
Evaluation of the regulatory response during SLIT with D_1_ManPrup3 or D_4_ManPrup3. (**A**) Median and interquartile ranges for percentages of CD11c^+^CD103^+^ cells (Left) and cells producing intracellular IL-10 (Right). (**B**) Median and interquartile ranges for percentages of regulatory CD4^+^CD25^+^FoxP3^+^ cells (Left) and cells producing intracellular IL-10 (Right). Significant differences (p < 0.05) are represented as * when compared to PBS treated group.

We further analyzed the influence of SLIT on the cellular regulatory response by analyzing changes in regulatory DCs (CD11c^+^CD103^+^) and CD4^+^ splenocytes (CD4^+^CD25^+^FoxP3^+^) (Fig. 4). No significant differences for regulatory DCs were found compared to PBS-treated mice, for any condition except for mice treated with D_4_ManPrup3 at 5 nM and after 5 w (Fig. 4A left). A significant increase in Tregs was observed for mice treated with D_1_ManPrup3 at 2 or 5 nM, and D_4_ManPrup3 at 2 nM at 1 w (Fig. 4B left). These increases disappeared at 5 w in mice treated with D_1_ManPrup3 at 5 nM. Interestingly, in mice treated with D_4_ManPrup3 at 5 nM a significant increase of both regulatory DCs and Tregs appeared at 5 w only.

The analysis of IL10-producing cells showed a significant increase in mice treated with D_1_ManPrup3 at 2 and 5 nM and with D_4_ManPrup3 at 2 nM in both DCs and CD4 cells at 1 w. These increases were maintained in CD4 cells but not in DCs for D_1_ManPrup3; in the case of mice treated with D_4_ManPrup3 the increase disappeared in both DCs and CD4 cells (Fig. 4 right).

## Discussion

Food allergy is becoming an important health problem for children and adults^1,21^. Current treatments, such as AIT show promise, but in many cases, such as LTP syndrome, better treatments are required^2^.

One potential factor for the current lack of AIT efficacy may be related to allergen concentration. However, given that complete proteins from the allergenic extracts are used in conventional AIT, higher concentrations might increase allergic reactions. Moreover, despite efforts towards standardization, large-scale production of vaccines using natural protein extracts can lead to differences between batches. Therefore, there is much interest in the development of new immunotherapy approaches, including modified food allergens or peptides. The use of longer contiguous overlapping peptides for cat allergy^22^ although promising in initial trials, did not achieve the clinical end points at phase II, with no differences compared to placebo^13^. It may be the case that this type of treatment requires the immunological system to be boosted by adding an adjuvant. To this end, adjuvants that interact with toll like receptors (TLRs), specifically with TLR9, have been used to shift the Th2 immune response towards a Th1/Treg balance in allergic rhinitis^23^ as well as FA^24^. Our group recently showed that the use of this ligand with a dendrimeric structure containing Prup3-T cell peptides induced protection from anaphylaxis in experimental models^15^. Similar results have also been found for peanut extract^16^.

In addition to TLRs, C-type lectin receptors represent another potential target on antigen presenting cells that can improve the uptake and presentation of different compounds modulating the immunological response, as has been demonstrated using gold nanoparticles and glycodendrimers^17,19^. In allergic diseases, mannan-conjugated allergoids have been shown to increase allergen uptake and induce splenic Foxp3^+^Treg cell production^25^. For this we designed a new synthetic strategy which confers an important advantage in that it uses glycodendritic platforms that can be functionalized easily using a thiol-ene reaction by a terminal cysteine present at one end of the peptide epitope. Here, we have put these elements together to create a novel SLIT approach for peach allergy, in which one or four Prup3 T-cell peptides are combined with mannose dendrons.

The results suggest that the optimal quantity of SLIT using D_1_ManPrup3 or D_4_ManPrup3 would be 2 nM, as this led to the most tolerant response, as measured by no changes in body temperature upon challenge, a decrease of Prup3-specific IgE and -IgG1 level and secreting cells as well as Prup3 specific-splenocyte CD4 proliferation and IL-4 production accompanied by an increase in IFN-γ and IL-10-producing splenocytes and Tregs. Compared to previous results from our group where CpG was used as adjuvant, the percentage of cases with tolerant responses was a little bit higher: 90% with D_1_ManPrup3 vs 80% with D_1_Prup3 + CpG, with similar results at *in vitro* levels^15^.

We also assessed the effect of SLIT with these GDPs on DCs since, as has been reported, AIT efficacy may directly correlate with the capability of SLIT to modulate DCs^13^. After treatment with D_1_ManPrup3 at 2 nM, we observed a significant decrease of IL-4, in parallel with an increase of IFN-γ and IL-10-producing DCs. This suggests that SLIT is modulating DCs to produce the specific signalling molecules needed to induce Tregs and thus supress the Th2 response^26^. It has been reported that changes in CD103^+^CD11c^+^DCs during AIT are related to the induction of antigen-specific Foxp3^+^Treg cells^27^, however although we observed increases in FoxP3^+^Tregs, no significant change in CD103^+^CD11c^+^DCs was found. This discrepancy could be due to the fact that CD103^+^CD11c^+^DCs from different lymph nodes were analyzed together, making it difficult to find population-specific changes. Further studies should be performed to identify whether only a subset of DCs, i.e. from the submandibular^27^ or mesenteric lymph nodes^28^, are modified by our system.

One of the main aims of SLIT as a FA treatment is to induce sustained unresponsiveness that persists after finishing treatment. However, to date only a handful of studies have addressed this aspect, and only for oral immunotherapy^29,30^. In animal models the protective effects induced by oral immunotherapy with egg white or ovomucoid were lost after 2 weeks^31^. Here, we found that only D_1_ManPrup3 at 2 nM induced the maintenance of tolerance five weeks after stopping SLIT, with 90% of the mice in this group not showing anaphylactic symptoms. Moreover, this was underlined by similar immunological changes to those obtained one week after finishing treatment. Importantly in our model, this sustained unresponsiveness was achieved without continued exposure to the allergen, which has previously been proposed by others as a requirement to maintain the unresponsive state^29^.

Regarding D_4_ManPrup3 at 2 nM, although beneficial effects were observed 1 week after treatment, these disappeared after 5 weeks, indicating a desensitization effect rather than sustained unresponsiveness. Previous work from our group investigating SLIT using dendrimeric structures with CpG as an adjuvant found that the tetrameric structure afforded no protection at all^15^. It may be that D_4_ManPrup3 is more effective in this current study due to the presence of mannose rather than CpG. The lesser effects of D_4_ManPrup3, compared to D_1_ManPrup3, could be due to the size of the nanostructure or differences in presentation, which may affect DC uptake and the induction of the immunosuppressive Th1/Treg responses^32^. Moreover although further studies are necessary, we have observed that these tetrameric compounds, both with CpG^15^ and with mannose (data not shown) have the capacity to cross-link at least two sIgE on the basophil surface, which is associated with an allergic or Th2 response.

One interesting finding is that D_1_ManPrup3 only induced a long-term beneficial effect when administered at 2 nM. This long term effect obtained with D_1_ManPrup3 at 2 nM was associated with the induction of FoxP3^+^Treg cells and IL-10 production, both of which are thought to be essential in tolerance induction^33,34^. Higher concentrations of 5 nM, induced a protective effect initially, however this disappeared after 5 weeks. This dose-dependent effect has been observed in 2 trials of intranasal AIT for rhinitis and rhinoconjunctivitis^35,36^ and in a study of AIT in a murine model of shrimp allergy^33^. This lower effect may be explained by the results of previous studies showing that a high concentration of antigen exposure is related with a Th2 polarization and a lack of FoxP3^+^Treg induction^37,38^

In conclusion, we have shown that SLIT with a monomeric GDP of Prup3, D_1_ManPrup3, induces protection, as defined by an absence of anaphylaxis symptoms, against the allergen, Prup3. This tolerance was associated with a decreased Th2 response (IgE, IgG1 and IL-4) and an increased Th1/Treg pattern (IFN-γ and IL-10). Moreover, the long-term sustained unresponsiveness persisted for at least five weeks after treatment and without the need to maintain regular contact with the allergen. Thus, D_1_ManPrup3 represents a promising new specific immunotherapy approach that does not require additional adjuvants and moreover, the molecular design and chemical route for access to the composition make it highly versatile and potentially adaptable to other types of allergens for which the chemical structure of the antigenic determinants are known.

## Methods

### Synthesis of GDPs including Prup3-epitope

The synthesis and characterization of GDPs (Fig. 1) is described in detail in the Supplementary Material (Figs S1–S5). Selected peptide includes the essential residues to induce immunological responses in human PBMCs, including T and B epitopes^39^.

### Sensitization and Immunotherapy procedures

The experimental procedures in animals were approved by the Animal Experimentation Ethics Committee of BIONAND, Malaga, Spain, (REF26072017) according to international standards of animal welfare and confirming that all experiments were performed in accordance with relevant guidelines and regulations. SLIT efficacy was assessed using a previously established murine model of peach allergy^15^. BALB/c mice (female, 4–5 weeks: Janvier Lab, Saint-Berthevin Cedex, France) were used, divided into 17 different groups (N = 10 per group) (Table 2). The protocols for sensitization, immunotherapy and challenge were as described^15,40^.

**Table 2:** Groups of mice included and treatment received during the study.

Group	Sensitized to Prup3	Treatment	D_n_ManPrup3 concentration in SLIT	Time of Prup3 challenge after SLIT (weeks)
1	No	—		1
2				5
3	Yes	PBS		1
4				5
5		D_1_ManPrup3	1 nmol	1
6				5
7			2 nmol	1
8				5
9			5 nmol	1
10				5
11		D_4_ManPrup3	1 nmol	1
12				5
13			2 nmol	1
14				5
15			5 nmol	1
16				5
17		Prup3	10 µg	1

#### Sensitization

Mice from groups 3–17 were anaesthetized and treated with 20 µg of natural Prup3 (Bial Laboratory, Zamudio, Spain) (optimal concentration after previous studies) and 20 ng of LPS (InvivoGen, San Diego, CA) in a final volume of 12 µl applied intranasally. This approach was chosen based on our previous experience by which only in combination with LPS, Prup3-specific anaphylaxis can be generated after nasal sensitisation^40^ since the respiratory tract has also been suggested in food sensitization^41,42^. The administration schedule was three consecutive days plus once a week for four weeks. The same schedule and volume were followed with PBS in mice of group 1 (Table 2).

#### Immunotherapy

At week 6, after sensitization, mice from groups 5–16 were treated sublingually with 10 µl of D_1_ManPrup3 or D_4_ManPrup3 (1, 2 or 5 nM/dose), weekly for eight weeks. For mice of group 3 and 4 we followed the same schedule but with 10 µl of PBS and for group 17 with Prup3 at 10 µg/mL (Table 2).

### *In vivo* assessment of tolerant response

The immunotherapy efficacy was assessed by determining the inhibition of the appearance of systemic anaphylaxis after challenge with Prup3 in one intraperitoneal dose (100 µg). This was evaluated by measuring change in body temperature with a rectal probe, and by assessing physical and behavioural symptoms (according to a scoring system) at weeks 14 and 18, 30–40 min (Fig. 2A). The most characteristic symptoms were inactivity, isolation from other animals, difficulty breathing and convulsions.

For further studies, blood from mice were obtained from the retro-orbital plexus on day 0 (basal) and at either week 14 or 18 after challenge, and sera were stored at −20 °C. After challenge and *in vivo* evaluation, mice were sacrificed by cervical dislocation and the spleen and lymph nodes were removed aseptically and teased to prepare a single-cell suspension for cellular analysis.

### Prup3-sIgE and -sIgG1 levels and secreting cells determination

The levels of Prup3-specific IgE and IgG1 were measured by ELISA as described (Rodriguez, Mascaraque, *et al*., 2017). The results are expressed as optical density.

The number of antibody-secreting cells was measured using an ELISpot assay as previously described^40^. The results are expressed as number of Ig secreting cells.

### Evaluation of the cellular response

Proliferative response of CD4 and CD8 spleen cells after immunotherapy was assessed by flow cytometry as described^15,40^. Results were expressed as proliferation index (PI) for CD4+ or CD8+ measured as the ratio: %CD4^+^CFSE_dim_ or %CD8^+^CFSE_dim_ in stimulated sample/%CD4^+^CFSE_dim_ or %CD8^+^CFSE_dim_ in non-stimulated sample and considered positive when the ratio was higher than 2.

The cytokine pattern was evaluated in spleen cells by determining intracellular cytokines using BD Cytofix/CytoPerm Fixation/Permeabilization solution kit (BD™ Biosciences) and staining with specific fluorochrome-conjugated mAbs, anti-IFNγ-APCCy7, ant-iIL-4-PE-Cy7A and anti-IL-10-PE (BD Pharmingen). Regulatory cells were defined as CD3^+^CD4^+^CD25^+^FoxP3^+^ using anti-CD25 and FoxP3 (BD Pharmingen). Results are expressed as percentages.

### Studies of dendritic cells and cytokine production

Dendritic cells were purified from axillary, cervical, inguinal and mesenteric lymph nodes by positive selection using CD11c microbeads (Miltenyi Biotec, Bergisch Gladbach, Germany). The CD11c^+^ fraction was incubated in the absence (negative control) or presence of Prup3 at 25 µg/mL at 37 °C and 5% of CO_2_.

In order to assess dendritic cells regulatory responses, after 48 hours of the culture, the cells were stained with specific fluorochrome-conjugated mAbs, anti-CD11c-APC and anti-CD103-FITC and phenotyped as described above. For intracellular cytokine detection, cells were fixed, stained and phenotyped, using anti-IFNγ-APCCy7, anti-IL-4-PE-Cy7A and anti-IL-10-PE (BD Pharmingen). Results are expressed as percentages.

### Statistical analysis

Data are presented as individual data values or mean and standard deviation (S.D.). For unrelated samples comparisons Mann-Whitney U, Kruskal-Wallis or χ^2^-test were used. Related samples were compared using the Wilcoxon signed-rank test. P-values lower than 0.05 were considered statistically significant.

## Supplementary information

Supporting Information

## Data Availability

The datasets generated during and/or analysed during the current study are available from the corresponding author on reasonable request.
