# Author Correction: Glycosylated nanostructures in sublingual immunotherapy induce long-lasting tolerance in LTP allergy mouse model

**DOI:** 10.1038/s41598-020-63855-2

**Published:** 2020-04-17

**Authors:** Maria J. Rodriguez, Javier Ramos-Soriano, James R. Perkins, Ainhoa Mascaraque, Maria J. Torres, Francisca Gomez, Araceli Diaz-Perales, Javier Rojo, Cristobalina Mayorga

**Affiliations:** 1 Allergy Research Group, Instituto de Investigación Biomédica de Málaga-IBIMA, Málaga, Spain; 2 Medicine Department, Universidad de Málaga-UMA, Málaga, Spain; 3 Glycosystems Laboratory, Instituto de Investigaciones Químicas (IIQ), CSIC - Universidad de Sevilla, Sevilla, Spain; 4 Allergy Clinical Unit, Hospital Regional Universitario de Málaga, Málaga, Spain; 5 Nanostructures for Diagnosing and Treatment of Allergic Diseases Laboratory, Centro Andaluz de Nanomedicina y Biotecnología-BIONAND, Málaga, Spain; 6 Center for Plant Biotechnology and Genomic (UPM-INIA), Madrid, Spain

Correction to: *Scientific Reports* 10.1038/s41598-019-40114-7, published online 11 March 2019

The Acknowledgements section in this Article is incomplete.

“The authors would like to thank Mrs Reyes Molina from the animal facility service at the Andalusian Centre for Nanomedicine & Biotechnology (BIONAND), Malaga, Spain. Mrs Ana Molina for the immunological analysis in IBIMA laboratories, Malaga, Spain and David Andreu from the Department of Experimental and Health Sciences, Universitat Pompeu Fabra of Barcelona for the synthesis of Prup3 T-cell peptide. The study was funded by the Institute of Health “Carlos III” of the Ministry of Economy and Competitiveness: PI12/02481, PI15/00559 and PI18/00288, RETICS ARADyAL (RD16/0006/0001, /0003, /0011) and Sara Borrell Program (CD14/00242). Andalusian Regional Ministry of Economy and Knowledge: CTS-7433), Nicolas Monardes Program (C-0044-2012 SAS2013) and the Spanish Ministry of Economy and Competitiveness (CTQ2014-52328-P, CTQ2017-86265-P and AGL2014-52395-C2-2) and a FPI grant (JRS). Grants were cofunded by the European Regional Development Fund (ERDF).”

should read:

“The authors would like to thank Mrs Reyes Molina from the animal facility service at the Andalusian Centre for Nanomedicine & Biotechnology (BIONAND), Malaga, Spain. Mrs Ana Molina for the immunological analysis in IBIMA laboratories, Malaga, Spain and David Andreu from the Department of Experimental and Health Sciences, Universitat Pompeu Fabra of Barcelona for the synthesis of Prup3 T-cell peptide. The study was funded by the Institute of Health “Carlos III” of the Ministry of Economy and Competitiveness co-founded by Fondo Europeo de Desarrollo Regional for the Thematic Networks and Co-operative Research Centres Una manera de hacer Europa: PI12/02481, PI15/00559 and PI18/00288, RETICS ARADyAL (RD16/0006/0001, /0003, /0011) and Sara Borrell Program (CD14/00242). Andalusian Regional Ministry of Health, Nicolas Monardes Program (C-0044- 2012 SAS2013). Andalusian Regional Ministry of Economy and Knowledge: CTS-7433), and the Spanish Ministry of Economy and Competitiveness (CTQ2014-52328-P, CTQ2017-86265-P and AGL2014-52395-C2-2) and a FPI grant (JRS). Grants were cofunded by the European Regional Development Fund (ERDF).”

