# Supplementary material for: Glycosylated nanostructures in sublingual immunotherapy induce long-lasting tolerance in LTP allergy mouse model
Source: Sci Rep. 2019 Mar 11;9:4043. doi: 10.1038/s41598-019-40114-7 (PMC6411722; doi:10.1038/s41598-019-40114-7)
Supplement: Supplementary file 1 — Supporting Information [file 41598_2019_40114_MOESM1_ESM.docx]

# Title: Glycosylated nanostructures in sublingual immunotherapy induce long-lasting tolerance in LTP allergy mouse model

**Authors:** Maria J Rodriguez, BsC^a#^, Javier Ramos-Soriano, BsC^b#^, James R Perkins, PhD^a^, Ainhoa Mascaraque, PhD^b^, Maria J Torres, MD, PhD^c^, Francisca Gomez, MD, PhD^c^, Araceli Diaz-Perales, PhD^d^, Javier Rojo, PhD^b^*, Cristobalina Mayorga, PhD^a,c^*

**Affiliation:** ^a^Research Laboratory, IBIMA, Regional University Hospital of Malaga, UMA, Malaga, Spain, ^b^Glycosystems Laboratory, Instituto de Investigaciones Químicas (IIQ), CSIC - Universidad de Sevilla, Sevilla, Spain. ^c^Allergy Unit, IBIMA, Regional University Hospital of Malaga, UMA, Malaga, Spain, ^d^Center for Plant Biotechnology and Genomic (UPM-INIA), Madrid, Spain,

# and * These authors contributed equally in this manuscript

**Corresponding author:**

PhD. Cristobalina Mayorga Mayorga

Research Laboratory-Allergy Service, Pavilion 5, basement

Hospital Regional Universitario de Malaga. Plz Hospital Civil. Málaga, Spain

Tel: +34 951290224. FAX: +34 951290302; E-mail: [lina.mayorga@ibima.eu](mailto:lina.mayorga@ibima.eu)

1. **Synthesis and Characterization of GDPs and the corresponding synthetic intermediates**

**Figure S1.** Synthetic scheme for the preparation of GDPs **6** and **7**.

**General protocol for glyco-maleimide dendrons synthesis 1-2**.

A solution of CuBr (5.10 μmol) and TBTA (10.20 μmol) in DMSO (166 μL) was added to a solution of the maleimido derivative 3[[1](#_ENREF_1)] or the tetravalent dendron 4[[2](#_ENREF_2)] (7.65 μmol) in DMSO (83 μL). Subsequently, a solution of the corresponding glycodendron 5^2^(5.10 μmol) in sodium phosphate buffer (16 mm, pH 7.4, 249 μL) was added to the former solution. After shaking for aprox. 30 min at room temperature (the progression of the reaction was monitored by analytical RP-HPLC), the reaction mixture was directly purified by semipreparative RP-HPLC to give the target compounds 1-2 (Figures S2-S3).

***Glyco-maleimide dendron D_1_Man_9_ 1***

Following the general procedure, using compound **5** and glycodendron **7** as starting materials, and purification by semipreparative RP-HPLC (column C_8_ and the elution with a linear gradient from 12 to 50% of solvent B into A over 30 min, at 3 mL/min flow rate (A: H_2_O (0.05% TFA), B: ACN (0.1% TFA))), compound **1** (6.8 mg, 1.63 μmol, 32%) was obtained as a white solid. Characterization data are shown in Figure S2.

^1^H-RMN (400 MHz, D_2_O) 8.04 (s, 10H, H_triazole_), 8.02 (s, 3H, H_triazole_), 6.74 (s, 2H, H_maleimide_), 5.13 (s, 2H, OCOCH_2_C_triazole_), 4.79 (under D_2_O, 9H, H-1), 4.69 (m, 8H, O_linker_CH_2_CH_2_N), 4.64 (m, 18H, OCH_2_CH_2_N), 4.53 (s, 24H, OCH_2_C_triazole_), 4.48 (m, 8H, O_linker_CH_2_CH_2_N), 4.09 (m, 9H, OCHHCH_2_N), 3.98-3.87 (m, 17H, OCHHCH_2_N, CCH_2_OCO), 3.85 (m, 9H, H-2), 3.77-3.55 (m, 38H, H-3, H-5, H-6, CH_2_CH_2_N_maleimide_), 3.40 (s, 18H, CCH_2_O), 3.37 (s, 6H, CCH_2_O), 3.08 (m, 9H, H-4), 2.65 (t, 2H, J_H,H_ = 6.6, CH_2_CH_2_N_maleimide_), 2.51 (br s, 16H, CH_2,succ._); ^13^C-RMN (100 MHz, D_2_O) 173.6-173.4 (CO_succ_), 172.6 (CO), 172.2 (CO_maleimide_), 144.4 (C_triazole_), 134.3 (CH_maleimide_), 125.5 (CH_triazole_), 99.5 (C-1), 72.8 (C-4), 70.4 (C-3), 69.9 (C-2), 68.1 (CCH_2_O), 67.9 (CCH_2_O), 66.4 (C-5), 65.4 (OCH_2_CH_2_N), 63.6 (OCH_2_C_triazole_), 63.1 (O_linker_CH_2_CH_2_N, CCH_2_OCO), 60.7 (C-6), 57.4 (OCOCH_2_C_triazole_), 50.0 (OCH_2_CH_2_N), 49.1 (O_linker_CH_2_CH_2_N), 43.9 (CCH_2_O), 33.4 (CH_2_CH_2_N_maleimide_), 32.6 (CH_2_CH_2_N_maleimide_), 28.7, 28.6 (CH_2,succ._); MALDI-ToF m/z calcd. for C_162_H_244_N_40_O_86_: 4127.9; found: 4151.6 [M+Na]^+^; HPLC t_R_ = 8.39 min (Column: analytical C_8_; A: H_2_O (0.05 % TFA), B: Acetonitrile (0.1 % TFA), 15 to 25% linear gradient of B into A over 15 min, F = 1 mL/min).

***Glyco-maleimide dendron D_4_Man_9_ 2***

Following the general procedure, using compound **4** and glycodendron **5** as starting materials, and purification by semipreparative RP-HPLC (column C_18_ and the elution with a linear gradient from 12 to 50% of solvent B into A over 30 min, at 3 mL/min flow rate (A: H_2_O (0.05% TFA), B: ACN (0.1% TFA)), compound **2** (9.0 mg, 1.84 μmol, 36%) was obtained as a white solid. Characterization data are shown in Figure S3.

^1^H-NMR (400 MHz, D_2_O) 8.10 (s, 1H, H_triazole_), 8.04 (s, 9H, H_triazole_), 8.01 (s, 3H, H_triazole_), 6.82 (s, 8H, H_maleimide_), 5.23 (s, 2H, OCOCH_2_C_triazole_), 4.79 (under D_2_O, 9H, H-1), 4.70 (m, 8H, O_linker_CH_2_CH_2_N), 4.64 (m, 18H, OCH_2_CH_2_N), 4.53 (s, 24H, OCH_2_C_triazole_), 4.49 (m, 8H, O_linker_CH_2_CH_2_N), 4.22 (AB system, 4H, C_a_CH_2_O), 4.15-4.02 (m, 17H, OCHHCH_2_N, C_b_CH_2_O), 3.99-3.87 (m, 17H, OCHHCH_2_N, CCH_2_OCO), 3.85 (m, 9H, H-2), 3.78-3.56 (m, 44H, H-3, H-5, H-6, CH_2_CH_2_N_maleimide_), 3.41 (br s, 24H, CCH_2_O), 3.09 (m, 9H, H-4), 2.65 (t, 8H, J_H,H_ = 6.4, CH_2_CH_2_N_maleimide_), 2.52 (br s, 16H, CH_2,succ._), 1.22 (s, 3H, (CH_3_)_a_), 1.11 (s, 6H, (CH_3_)_b_); ^13^C-NMR (100 MHz, D_2_O) 173.5-173.1 (CO_succ_), 172.3 (CO), 172.2 (CO_maleimide_), 144.4 (C_triazole_), 144.2 (C_triazole_), 134.5 (CH_maleimide_), 125.3 (CH_triazole_), 125.1 (CH_triazole_), 99.6 (C-1), 72.8 (C-4), 70.5 (C-3), 69.9 (C-2), 68.2 (CCH_2_O), 68.0 (CCH_2_O), 66.4 (C-5), 65.9 (OC_a_H_2_, OC_b_H_2_), 65.4 (OCH_2_CH_2_N), 63.8 (OCH_2_C_triazole_), 63.6 (OCH_2_C_triazole_), 63.1 (O_linker_CH_2_CH_2_N, CCH_2_OCO), 60.7 (C-6), 57.4 (OCOCH_2_C_triazole_), 50.0 (OCH_2_CH_2_N), 49.1 (O_linker_CH_2_CH_2_N), 46.5 (C_q,a_), 46.1 (C_q,b_), 43.9 (CCH_2_O), 33.3 (CH_2_CH_2_N_maleimide_), 32.5 (CH_2_CH_2_N_maleimide_), 28.7, 28.6 (CH_2,succ._), 16.9 (C_b_H_3_), 16.8 (C_a_H_3_); MALDI-ToF m/z calcd. for C_198_H_283_N_43_O_104_: 4926.8; found: 4949.9 [M+Na]^+^; HPLC t_R_ = 10.82 min (Column: analytical C_18_; A: H_2_O (0.05 % TFA), B: Acetonitrile (0.1 % TFA), 12 to 50% linear gradient of B into A over 15 min, F = 1 mL/min).

**General protocol for the preparation of GDPs 6-7**.

Another solution of peptide Pru p 3 SH[[3](#_ENREF_3)] (1.1 eq. for each maleimide) in H_2_O (567 µL) was added to a solution of glyco-maleimide dendron **1**-**2** (0.75 µmol, 1 eq.) in sodium phosphate buffer (50 mm, pH 7.4, 1134 µL). After shaking for aprox. 30 min at room temperature (the progression of the reaction was monitored by analytical RP-HPLC), the reaction mixture was lyophilized, and then purified by semipreparative RP-HPLC to give target compounds **6**-**7** (Figures S4-S5).

***D_1_ManPrup3 6***

Following the general procedure, using glycodendron **1** as starting material, and purification by semipreparative RP-HPLC (column C_18_ and elution with a linear gradient from 12 to 30% of solvent B into A over 30 min, at 3 mL/min flow rate (A: H_2_O (0.05% TFA), B: ACN (0.1% TFA)), compound **6** (3.7 mg, 0.58 μmol, 77%) was obtained as a white solid. Characterization data are shown in Figure S4.

ESI-MS (deconvoluted) m/z calcd. for C_251_H_398_N_76_O_117_S: 6384.42; found: 6385.02 [M]^+^; HPLC t_R_ = 9.54 min (Column: analytical C_18_; A: H_2_O (0.05 % TFA), B: Acetonitrile (0.1 % TFA), 12 to 30% linear gradient of B into A over 15 min, F = 1 mL/min).

***D_4_ManPrup3 7***

Following the general procedure, using glycodendron **2** as starting material, and purification by semipreparative RP-HPLC (column C_18_ and elution with a linear gradient from 15 to 40% of solvent B into A over 30 min, at 3 mL/min flow rate (A: H_2_O (0.05% TFA), B: ACN (0.1% TFA)), compound **7** (6.6 mg, 0.47 μmol, 63%) was obtained as a white solid. Characterization data are shown in Figure S5.

ESI-MS (deconvoluted) m/z calcd. for C_554_H_899_N_187_O_228_S_4_: 13955.61; found: 13955.48 [M]^+^; HPLC t_R_ = 7.17 min (Column: analytical C_18_; A: H_2_O (0.05 % TFA), B: Acetonitrile (0.1 % TFA), 15 to 40% linear gradient of B into A over 15 min, F = 1 mL/min).

**A) HPLC chromatogram**

**B) MALDI-ToF**

**Figure S2**. **A)** HPLC monitoring of the click chemistry reaction for obtaining compound **1** and the peak corresponding to the same compound after HPLC purification (Left). **B)** MALDI-ToF for compound **1**: MW calculated 4127.9 (M+) Dalton; Found 4151.6 Dalton (M+Na)

**A) HPLC chromatogram**

**B) MALDI-ToF**

**Figure S3**. **A)** HPLC monitoring of the click chemistry reaction for obtaining compound **2** and the peak corresponding to the same compound after HPLC purification (Left). **B)** MALDI-ToF for compound **2**: MW calculated 4926.8 (M+) Dalton; Found 4949.9 Dalton (M+Na)

**A) HPLC chromatogram**

**B) ESI-MS (deconvoluted)**

**Figure S4. A)** HPLC monitoring of the thiol-ene reaction for obtaining compound **6** and the peak corresponding to the same compound after HPLC purification (left). **B)** ESI-MS (deconvoluted) for compound **6**: MW calculated 6384.4 (M+) Dalton; Found 6385.0 Dalton (M+H)

**A) HPLC chromatogram**

**B) ESI-MS (deconvoluted)**

**Figure S5**. **A)** HPLC monitoring of the thiol-ene reaction for obtaining compound **7** and the peak corresponding to the same compound after HPLC purification (left). **B)** ESI-MS (deconvoluted) for compound **7**: MW calculated 13955.6 (M+) Dalton; Found 13955.5 Dalton (M+H).

**References**

1. Song, H.Y., et al., *Practical synthesis of maleimides and coumarin-linked probes for protein and antibody labelling via reduction of native disulfides.* Org Biomol Chem, 2009. **7**(17): p. 3400-6.

2. Kowalczyk, W., et al., *Convergent Synthesis of Glycodendropeptides by Click Chemistry Approaches.* Eur. J. Org. Chem, 2012. **24**: p. 4565–4573.

3. Rodriguez, M.J., et al., *Pru p 3-Epitope-based sublingual immunotherapy in a murine model for the treatment of peach allergy.* Mol Nutr Food Res, 2017. **61**(10).
